# Usual care for youth with autism spectrum disorder: Community-based providers’ reported familiarity with treatment practices

**DOI:** 10.3389/fpsyt.2022.923025

**Published:** 2022-07-26

**Authors:** Matthew D. Lerner, Cynthia E. Brown, Aksheya Sridhar, Jessica E. Tschida, Peter Felsman, Erin J. Libsack, Connor M. Kerns, Lauren J. Moskowitz, Latha Soorya, Allison Wainer, Elizabeth Cohn, Amy Drahota

**Affiliations:** ^1^Department of Psychology, Stony Brook University, Stony Brook, NY, United States; ^2^School of Graduate Psychology, Pacific University, Hillsboro, OR, United States; ^3^Department of Psychology, Michigan State University, Lansing, MI, United States; ^4^Department of Social Work, Northern Michigan University, Marquette, MI, United States; ^5^Department of Psychology, The University of British Columbia, Vancouver, BC, Canada; ^6^Department of Psychology, St. John’s University, Queens, NY, United States; ^7^Department of Psychiatry, Rush University Medical Center, Chicago, IL, United States; ^8^Hunter-Bellevue School of Nursing, City University of New York, New York, NY, United States

**Keywords:** autism spectrum disorder, psychosocial treatment, community-based, usual care, familiarity, treatment practices, youth

## Abstract

**Objective:**

To examine patterns and predictors of familiarity with transdisciplinary psychosocial (e.g., non-pharmacologic) practices for practitioners treating youths with autism spectrum disorder (ASD) in the United States.

**Method:**

Practitioners (*n* = 701) from behavioral, education, medical, and mental health backgrounds who worked with youth (ages 7–22) with ASD completed the Usual Care for Autism Survey, which assessed provider demographics and self-reported familiarity with transdisciplinary treatment practices for the most common referral problems of ASD. We examined relations between provider-, setting-, and client-level characteristics with familiarity of key groups of the treatment practices (practice sets). Practice sets were identified using exploratory factor analysis (EFA), and demographic predictors of practice subsets were examined using generalized estimating equations (GEE).

**Results:**

The EFA yielded a three-factor solution: (1) *environmental modifications/antecedent strategies*; (2) *behavior analytic strategies*; and (3) *cognitive strategies*, with overall familiarity ranked in this order. Medical providers indicated the least familiarity across disciplines. More experience with ASD and treating those with intellectual disabilities predicted greater familiarity with only *environmental modifications/antecedent strategies* and *behavior analytic*, but not *cognitive strategies*. Experience treating low SES clients predicted familiarity with *environmental modification* and *behavior analytic strategies* while experience treating high SES clients predicted familiarity with *behavior analytic* and *cognitive strategies*.

**Conclusion:**

This is the first study to identify transdisciplinary, interpretable sets of practices for treating youth with ASD based on community providers’ reported familiarity. Results highlight factors associated with familiarity with practice sets, which is essential for mapping practice availability, and optimizing training and dissemination efforts for youth with ASD.

## Introduction

The prevalence of autism spectrum disorder (ASD) continues to rise in the United States, with current estimates indicating that ASD affects 1 in 44 children (1). Due to the chronicity and severity of ASD core deficits and co-occurring challenges, youth with ASD present for mental health services at higher rates than neurotypical youth (2, 3), particularly for treatment of social skills deficits, externalizing symptoms, and anxiety, which are the most common referring problems for school-age youth with ASD (4–6). There exist many effective behavioral and medical interventions for addressing core and associated symptoms of ASD (7, 8); however, there is still a well-known gap between treatment access and need (the treatment-access gap) for youth with ASD and their families (4, 8–10). This gap is often seen in usual care settings (e.g., community mental and behavioral health clinics, schools, private outpatient therapy clinics) that provide services to school-age youth and adolescents with ASD (11–15).

Although there are many barriers to reducing the treatment-access gap, one centrally important barrier is limited provider familiarity with treatment practices (16). Familiarity with treatments permits providers to perform two core professional roles: (1) to select and deliver appropriate direct services, and/or (2) to determine appropriate referrals. Indeed, such familiarity is related to self-reported use, referral, and recommendation of interventions for youth with ASD (4, 17). When providers are unfamiliar with treatment practices important to the care of a condition, they limit their ability to perform either role—that is, familiarity is a necessary (if not sufficient) condition for increasing service access.

Providers’ training and practice backgrounds are likely one factor that facilitates familiarity with ASD-specific treatment practices (17–19). For example, service providers with educational versus medical training backgrounds may reasonably differ in exposure to common practices used in clinical settings (20, 21). However, clinical care guidelines for ASD necessitate a multi-disciplinary model integrating educational, behavioral, medical, and allied health models (18, 22, 23). Similarly, ASD providers treat individuals across a wide range of cognitive functioning, with familiarity with specific practices potentially varying as a function of client intellectual disability; for example, cognitive strategies versus some behavioral strategies may be used differentially as a function of cognitive ability. Understanding the factors that predict familiarity with treatments used in the care of individuals with ASD has potential to identify gaps in knowledge and ultimately provider training, education and collaboration, with potential to improve quality and access to care (22). Indeed, familiarity may also mediate other access-related factors, such as availability of services. That is, while services may exist and be available in a given region, if a family’s clinician or provider is not aware of or familiar with them, they will in turn not refer to them.

This study offers the first comprehensive picture of the current landscape of provider familiarity with treatments for youth with ASD in usual care settings in the United States, across all disciplines that regularly treat this population. Specifically, this study sought to: (a) identify treatment practice sets with which providers across disciplines report familiarity (using common language that can be understood by practitioners regardless of discipline, rather than discipline-specific jargon) in treating youth with ASD; (b) determine which provider-level (discipline, educational attainment, years providing services, practice settings) and client-level [presence of client intellectual disability (ID) and client SES] characteristics predict familiarity with treatment practice sets for youth with ASD.

## Materials and methods

### Participants

Participants targeted for recruitment included United States community-based behavioral, educational, medical, mental health, allied health, and other providers treating anxiety, externalizing behaviors, or social skills deficits in youth ages 7–22 years old with ASD during the year prior to recruitment. As outlined in the original protocol paper (8), this age range was selected because it represents the broadest possible window for treating school-age and teenage youth with ASD who may still be seen in the school system. Prior to age 7, some youth with ASD may still be transitioning to formal school-age classrooms, may not have developed some of the challenges that would indicate they may be referred for treatment (e.g., anxiety; school refusal), and so may not yet be accessing services designed to address the core challenge domains that are the focus of this project. After age 22, youth with autism will have transitioned out of school-based services. Participants were eligible if they completed the screening questionnaire identifying themselves as providing services to youth with ASD (see Table 1), provided an email address, and were located within 150 miles of one of the five academic sites (Drexel University, Rush University Medical Center, St. John’s University, Stony Brook University, and San Diego State University). This radius was identified because this project aimed to maximize the likelihood of including authentic participants who worked in discrete regions with youth with ASD. This design approach was selected to maximize interpretability and clarity of results. The identified geographic radius for each site allowed each site to leverage contact networks and distribute through established community channels, in hopes of achieving this maximization goal. As such, participants were recruited through existing regional networks and affiliate agencies, followed by snowball sampling. In total, 1,827 screening surveys were completed. Of these, 1,231 provider email addresses were supplied to Princeton Survey Research Associates International (PSRAI), an independent survey firm contracted to collect the online survey data. Of the 1,231 providers recruited to participate, 701 providers (56.9%) completed the study survey (see Data Screening below for final sample used in the present analyses). Data were collected from July 10 to October 6, 2017.

**TABLE 1:** Participant demographics.

Variables	Sample-wise familiarity: *M*, SD	Total sample	Drexel University	Rush University Medical Center	St. John’s University	San Diego State University	Stony Brook University
		*n* (%)	*n* (%)	*n* (%)	*n* (%)	*n* (%)	*n* (%)
		672 (100%)	158 (23.4%)	166 (24.6%)	159 (23.6%)	129 (19.1%)	62 (9.2%)
	Discipline***						
Allied health^a^	3.22,0.48	146 (21.7%)	35 (22.2%)	35 (21.1%)	22 (13.8%)	38 (29.5%)	16 (25.8%)
Behavioral^b^	3.29,0.56	112 (16.6%)	24 (15.2%)	41 (24.7%)	16 (10.1%)	27 (20.9%)	4 (6.5%)
Education^c^	3.23,0.46	156 (23.1%)	30 (19%)	26 (15.7%)	73 (45.9%)	19 (14.7%)	8 (12.9%)
Medical^d^	2.57,0.59	63 (9.3%)	14 (8.9%)	30 (18.1%)	9 (5.7%)	7 (5.4%)	3 (4.8%)
Mental health^e^	3.15,0.60	126 (18.7%)	39 (24.7%)	22 (13.3%)	22 (13.8%)	19 (14.7%)	24 (38.7%)
Other^f^	3.42,0.53	71 (10.5%)	16 (10.1%)	12 (7.2%)	17 (10.7%)	19 (14.7%)	7 (11.3%)
	**Educational Attainment**						
Less than 4-year	2.94,0.68	13 (1.9%)	3 (1.9%)	3 (1.8%)	2 (1.3)	3 (2.3%)	2 (3.2%)
4-year degree	3.15,0.47	78 (11.6%)	23 (14.6%)	29 (17.5%)	8 (5%)	13 (10.1%)	5 (8.1%)
Master’s degree	3.22,0.55	453 (67.3%)	95 (60.1%)	106 (63.9%)	117 (73.6%)	100 (77.5%)	35 (56.5%)
Doctoral degree	3.10,0.65	130 (19.3%)	37 (23.4%)	28 (16.9%)	32 (20.1%)	13 (10.1%)	20 (32.3%)
							
	Years working with ASD***						
0–10 years	3.24,0.53	364 (54%)	97 (61.4%)	78 (47%)	97 (61%)	65 (50.4%)	27 (43.5%)
11–20 years	3.05,0.61	247 (36.6%)	44 (27.8%)	80 (48.2%)	47 (29.6%)	53 (41.1%)	23 (37.1%)
20+ years	3.39,0.47	62 (9.2%)	17 (10.8%)	8 (4.8%)	15 (9.4%)	10 (7.8%)	12 (19.4%)
Missing		1 (0.1%)	0	0	0	1 (0.8%)	0
	**Treatment settings**						
1 setting	3.17,0.53	434 (64.4%)	102 (64.6%)	95 (57.2%)	113 (71.1%)	85 (65.9%)	39 (62.9%)
2 + settings	3.21,0.60	240 (35.6%)	56 (35.4%)	71 (42.8%)	46 (28.9%)	44 (34.1%)	23 (37.1%)
	**Treat co-occurring ID**						
Never/Rarely	3.04,0.59	54 (8.0%)	13 (8.2%)	9 (5.4%)	16 (10.1%)	8 (6.2%)	8 (12.9%)
Sometimes	3.21,0.53	348 (51.6%)	88 (55.7%)	100 (60.2%)	65 (40.9%)	69 (53.5%)	26 (41.9%)
Frequently	3.18,0.61	247 (36.6%)	53 (33.5%)	54 (32.5%)	65 (40.9%)	47 (36.4%)	28 (45.2%)
Unsure/Don’t know	3.07,0.51	25 (3.7%)	4 (2.5%)	3 (1.8%)	13 (8.2%)	5 (3.9%)	0
	High SES**						
No	3.13,0.58	414 (61.4%)	101 (63.9%)	110 (66.3%)	103 (64.8%)	60 (46.5%)	40 (64.5%)
Yes	3.27,0.54	260 (38.6%)	57 (36.1%)	56 (33.7%)	56 (35.2%)	69 (53.5%)	22 (35.5%)
	Low SES***						
No	3.05,0.58	297 (44.1%)	48 (30.4%)	90 (54.2%)	77 (48.4%)	59 (45.7%)	23 (37.1%)
Yes	3.29,0.53	377 (55.9%)	110 (69.6%)	76 (45.8%)	82 (51.6%)	70 (54.3%)	39 (62.9%)

**p = 0.001, ***p < 0.001.

^a^Comprised of speech and language therapists, occupational therapists, and physical therapists.

^b^Comprised of behavioral therapists, behavioral analysts, behavioral technicians, etc.

^c^Comprised of special educators, general educators, school psychologists, etc.

^d^Comprised of psychiatrists, neurologists, health practitioners, etc.

^e^Comprised of social workers, psychologists, counselors, etc.

^f^Comprised of managers, administrators, support workers, other disciplines, multiple disciplines, and unknown.

### Procedure

Each academic site obtained institutional IRB approval for the study procedures and conducted recruitment activities. Each eligible respondent (determined by screening questionnaire) was emailed invitations and email reminders containing a unique URL link to access the study survey by PSRAI. Respondents who completed the survey were paid a $40 honorarium for their participation. Data were de-identified by PSRAI and provided to the academic site investigators in Fall 2017.

### Measure

#### Usual care for youth with autism survey (7, 8)

The web-based survey was comprised of demographic questions as well as an inventory of 55 treatment practices derived through a literature review and two-round Delphi poll of expert ASD providers from multiple disciplines (7, 8). This process involved identifying practices referenced in the ASD intervention literature, providing them to the expert providers across disciplines, then, crucially, taking their feedback to both add and revise wording of items to ensure comprehensibility to their specific discipline and incorporating this into the next round of the poll to ensure transdisciplinarity. For example, expert providers across disciplines differentiated between *shaping* (reinforcing behaviors that are closer and closer “approximations” of a desired behavior until each approximation is mastered) and *reinforcement schedules* (controlling the timing and frequency of rewards in order to increase a desired behavior or decrease an undesired behavior), and identified practices that may be otherwise seen as less conventional, such as *non-contingent reinforcement or built in breaks*. Demographic items included provider- (i.e., race, ethnicity, discipline, years of experience working with individuals with ASD), setting- (i.e., region, practice setting, single vs. multiple practice settings), and client-level characteristics [i.e., SES, intellectual disability (ID)]. Notably, several of these characteristics (e.g., provider discipline, client SES) were non-mutually exclusive [e.g., a provider could be a Board Certified Behavioral Analyst (BCBA) and a Clinical Psychologist]. This study focused on the 32 practices that were implicated, based on expert consensus (7), in the treatment of the 3 identified key treatment areas: social skills, anxiety, and externalizing behavior. The remaining 23 practices were excluded from these analyses, as they were identified by experts as specific (e.g., implicated in social skills only), rather than transdiagnostic (implicated for all 3 presenting concerns). This process was undertaken because the present manuscript aimed to focus on familiarity with—and coherent sets of—practices for ASD regardless of treatment domains indicated by the participant. If a practice was identified to be specific only to the treatment of anxiety, for instance, this would likely skew its relation to other identified practices (i.e., participants would be more familiar with it if they treated anxiety or used other anxiety-related practices, and vice versa); thus, including only transdiagnostic practices allowed for identification of practices sets likely to be most generalized to all types of practitioners who work with youth with ASD. Participants rated their familiarity with each of these practices using a 4-point Likert rating scale ranging from 1 (*never heard of/not at all familiar*) to 4 (*very familiar with*).

### Data analysis plan

#### Data screening

We first examined the data structure to ensure that variables included in the data analysis plan met assumptions for factor analyses. Further, 27 participants were flagged by PSRAI due to concerns about participant fraud (24), and subsequently excluded due to identified differences in response patterns. The project team applied the Reflect, Expect, Analyze, Label Framework (REAL) framework (24) to identify fraudulent participants. Thus, the final sample included in the data analyses was 674 providers.

#### Descriptive statistics

A familiarity score for each respondent was calculated by averaging across responses on the 32 treatment practices. Analysis of variance (ANOVA) tests were performed to examine differences between provider-, client-, and setting-level demographic contrasts on familiarity. Because demographic variables were non-mutually exclusive, such contrasts represent conditional variables rather than person-level effects. When an ANOVA yielded a significant *F* statistic, multiple pairwise comparisons were conducted to determine the nature of between-groups differences with Bonferroni corrections to reduce the risk of Type 1 error in multiple comparisons.

#### Factor analysis and factor-level comparisons

Exploratory factor analyses using WLSMV estimation and goemin rotation were run in M+ to identify converging practice sets (i.e., factors) for familiarity ratings. Once the practice sets were identified, overall average familiarity scores for each of the practice sets were calculated and compared using a series of paired-samples t-tests.

#### Generalized estimating equations

Generalized estimating equations—able to account for possible non-independence of participant responses—were used to identify the provider- and client-level characteristics associated with familiarity with practice sets. Accounting for site differences, we predicted average familiarity with practice sets (DVs) by the independent variables: provider discipline, educational attainment, number of years worked with youth with ASD, treatment settings, and child characteristics, including co-occurring ID, high SES background, and low SES background. We ran each GEE utilizing unstructured and independent model structures and then selected the model with the lowest QIC model criterion coefficient.

## Results

### Descriptive statistics

Participants indicated they were broadly “somewhat familiar” (*M* = 3.18, *SD* = 0.57) with the overall treatment practices. At the provider level (Table 1), differences emerged based on provider discipline, with MDs endorsing less familiarity than all other groups (*p* < 0.001). Differences in familiarity were also observed for provider experience, with mid-experience providers (i.e., 11–20 years worked) reporting less familiarity than low-experience providers (i.e., 0-10 years worked) and high-experience providers (i.e., 21+ years worked; both *p* < 0.001). Regarding client SES, providers who worked with high SES clients and low SES clients reported higher average familiarity than those who reported not working with each of these SES groups (*p* = 0.001, *p* < 0.001, respectively).

### Factor analysis

Exploratory factor analysis yielded a three-factor solution with loading patterns that accounted for 75% of the variance in item responses (Table 2). The first factor, *environmental modifications/antecedent strategies*, included nine items, with an overall familiarity rating of 3.34 (*SD* = 0.60), explaining 61% of the overall variance in familiarity. The second factor, *behavior analytic strategies*, included 18 items, with an overall familiarity rating of 3.20 (*SD* = 0.62), explaining 8% of the overall variance. The third factor, *cognitive strategies*, included five items, and had an overall familiarity rating of 2.84 (*SD* = 0.71), explaining 6% of the overall variance. Providers reported greater familiarity with the *environmental modification*s/*antecedent strategies* practice set than with the *behavior analytic strategies* practice set, which in turn had greater familiarity than the *cognitive strategies* practice set.

**TABLE 2:** Practice sets representing familiarity with ASD treatment strategies.

Item number	Item name	Environmental modifications/antecedent strategies	Behavioral analytic strategies	Cognitive strategies
Q21	Choice making/providing choices	0.866*	0.052	–0.031
Q27	Embedding special interests in social interaction	0.614*	–0.013	0.149*
Q28	Environmental structuring	0.686*	0.215*	0.009
Q34	Games and activities that require social interaction	0.366*	0.106	0.299*
Q36	Homework	0.568*	–0.004	0.282*
Q40	Modeling or imitation	0.541*	0.256*	0.074
Q49	Priming	0.320*	0.177*	0.285*
Q63	Stories/vignettes	0.390*	0.345*	0.195*
Q70	Visual tools or supports	0.376*	0.345*	0.111*
Q25	Didactic teaching, social scripts, instructional learning	0.168*	0.423*	0.146*
Q26	Differential reinforcement	0.092	0.664*	–0.040
Q30	Extinction	–0.018	0.715*	0.040
Q31	Functional behavioral assessment (FBA)	0.040	0.741*	–0.050
Q32	Functional communication training (FCT)	0.138	0.649*	–0.140*
Q35	Gradual, graded, or habituated exposure/systematic desensitization	–0.003	0.462*	0.308*
Q43	Non-contingent reinforcement or built in breaks	0.136	0.612*	–0.030
Q44	Parent coaching	–0.086	0.528*	0.320*
Q45	Peer modeling or peer mentoring	0.200*	0.404*	0.208*
Q48	Positive reinforcement	0.410*	0.415*	0.021
Q50	Prompt fading	0.265*	0.630*	–0.017
Q51	Prompting	0.421*	0.462*	–0.012
Q53	Reinforcement schedules	0.019	0.781*	0.006
Q58	Self-management	0.000	0.447*	0.344*
Q59	Shaping	–0.159	0.863*	0.027
Q62	Stimulus control	–0.270	0.893*	0.014
Q65	Task analysis/chaining	0.128	0.650*	–0.019
Q68	Token economy	0.423*	0.465*	–0.030
Q41	Motivation by incorporating special interests into activities	0.279*	0.216*	0.368*
Q47	Performance feedback	0.228*	0.148*	0.407*
Q52	Psychoeducation	–0.067	0.006	0.748*
Q57	Self-awareness of bodily response	0.090	0.005	0.703*
Q61	Socratic discussions	0.284	–0.121	0.587*
% of Variance	75% (total)	61%	8%	6%
Number of Items	32	9	18	5

Factor loadings for the 32 practice items that loaded on at least one of three practice sets: environmental modifications/antecedent strategies, behavioral analytic strategies, and cognitive strategies. Final factor for each item is shaded.

*p < 0.05.

### Comparative GEE analyses

See Table 3 for selected model correlation structures and QIC coefficients. See Table 4 for a summary of the overall patterns of effects across contrast categories.

**TABLE 3:** Reported familiarity with practice sets.

	χ^2/^ Mean	SE	95% C.I.	α
***Familiarity with practice set (1) Environmental modifications/antecedent strategies***				
Independent GEE correlation model structure utilized (QIC = 239.51)				
**Discipline**	**250.9**			***p* < 0.001**
Allied health^a^	3.36	0.026	3.31–3.41	d < e, b, a, c < f
Behavioral provider^b^	3.28	0.063	3.16–3.40	
Education^c^	3.36	0.035	3.29–3.43	
Medical^d^	2.54	0.103	2.33–2.74	
Mental health^e^	3.16	0.16	2.84–3.47	
Other^f^	3.49	0.031	3.43–3.55	
**Educational Attainment**	**8.51**			***p* = 0.037**
Less than 4-year degree^g^	3	0.119	2.77–3.24	g, h < j, i
4-year degree^h^	3.18	0.046	3.09–3.27	g, h, j < i
Master’s degree^i^	3.31	0.021	3.27–3.36	g, h < j, i
Doctoral degree^j^	3.28	0.069	3.15–2.42	g < h, j, i
**Years working with ASD**	**30.28**			***p* < 0.001**
0-10 years^k^	3.21	0.038	3.13–3.28	l < k < m
11-20 years^l^	3.03	0.073	2.88–3.17	
21 + ^m^	3.36	0.049	3.26–3.45	
**Treatment Settings**	0.29			*NS*
1 setting	3.18	0.051	3.08–3.28	
2 + settings	3.22	0.052	3.11–3.32	
**Treat Co-occurring ID**	**14.99**			***p* = 0.002**
Unsure/Don’t know*^n^* Never/Rarely^o^	3.12	0.076	2.98–3.27	n, o, q < p
Sometimes*^p^*	3.17	0.045	3.08–3.26	n, o, q, p
Frequently*^q^*	3.28	0.047	3.19–3.37	n, q < o, p
	3.22	0.036	3.15–3.29	n, o, q < p
**High SES**	0.98			*NS*
No	3.17	0.033	3.11–3.24	
Yes	3.22	0.053	3.11–3.32	
**Low SES**	**8.78**			***p* = 0.003**
No	3.1	0.049	3.01–3.20	
Yes	3.29	0.051	3.19–3.39	
***Familiarity with Practice Set (2) Behavioral Analytic Strategies***				
Independent GEE correlation model structure utilized (QIC = 253.27)				
**Discipline**	**501.61**			***p* < 0.001**
Allied health^a^	3.03	0.057	2.92–3.15	d < e, a, c < b, f
Behavioral provider^b^	3.33	0.083	3.16–3.49	d, e, a < c, b, f
Education^c^	3.14	0.074	3.00–3.29	d < e, a, c, b < f
Medical^d^	2.43	0.126	2.19–2.68	d < e, a, c, b, f
Mental health^e^	2.91	0.123	2.67–3.15	d < e, a, c < b, f
Other^f^	3.35	0.059	3.23–3.47	d, e, a, c < b, f
**Educational Attainment**	**27.99**			***p* < 0.001**
Less than 4-year degree^g^	2.71	0.202	2.31–3.11	g, h < i, j
4-year degree^h^	2.95	0.028	2.89–3.00	
Master’s degree^i^	3.22	0.036	3.15–3.29	
Doctoral degree^j^	3.26	0.085	3.09–3.42	
**Years working with ASD**	**18.09**			***p* < 0.001**
0-10 years^k^	3.01	0.034	2.95–3.08	l < k < m
11-20 years^l^	2.87	0.063	2.75–2.99	
21 + ^m^	3.22	0.074	3.07–3.36	
***Familiarity with Practice Set (2) Behavioral Analytic Strategies***				
Independent GEE correlation model structure utilized (QIC = 253.27)				
**Treatment Settings**	3.01			*NS*
1 setting	2.99	0.045	2.90–3.07	
2 + settings	3.08	0.059	2.97–3.20	
**Treat Co-occurring ID**	**37.04**			***p* < 0.001**
Unsure/Don’t know*^n^*	2.99	0.072	2.85–3.13	n, o, p < q
Never/Rarely^o^	2.91	0.084	2.75–3.08	n, o, p, q
Sometimes*^p^*	3.08	0.06	2.96–3.20	n, o, p, q
Frequently*^q^*	3.14	0.057	3.03–3.26	n < o, p, q
**High SES**	**5.87**			***p* = 0.015**
No	2.99	0.043	2.91–3.08	
Yes	3.07	0.051	2.97–3.17	
**Low SES**	**8.45**			***p* = 0.004**
No	2.95	0.049	2.85–3.05	
Yes	3.12	0.056	3.01–3.23	
***Familiarity with Practice Set (3) Cognitive strategies***				
Independent GEE correlation model structure utilized (QIC = 333.91)				
**Discipline**	**101.67**			***p* < 0.001**
Allied health^a^	2.71	0.074	2.56–2.85	d, b < c, a, f < e
Behavioral provider^b^	2.53	0.062	2.41–2.65	d < b, c, f < a, e
Education^c^	2.62	0.087	2.45–2.79	d < b, c, a, f, e
Medical^d^	2.2	0.143	1.92–2.48	d < b, c, a, f, e
Mental health^e^	2.83	0.135	2.56–3.09	d, b < c, a, f, e
Other^f^	2.74	0.087	2.57–2.91	d < b, c, a, f, e
**Educational Attainment**	**12.79**			***p* = 0.005**
Less than 4-year degree^g^	2.21	0.216	1.79–2.63	g, h < i, j
4-year degree^h^	2.58	0.042	2.50–2.66	g, h < i, j
Master’s degree^i^	2.7	0.045	2.61–2.79	g, h < i < j
Doctoral degree^j^	2.93	0.081	2.77–3.09	g, h, i < j
**Years working with ASD**	**4.92**			*NS*
0-10 years^k^	2.68	0.047	2.59–2.77	
11-20 years^l^	2.55	0.045	2.46–2.63	
21 + ^m^	2.59	0.102	2.39–2.79	
**Treatment Settings**	**1.84**			*NS*
1 setting	2.56	0.071	2.42–2.70	
2 + settings	2.65	0.049	2.55–2.74	
**Treat Co-occurring ID**	**40.48**			***p* < 0.001**
Unsure/Don’t know*^n^*	2.29	0.07	2.15–2.43	n < q, o, p
Never/Rarely^o^	2.7	0.113	2.48–2.92	
Sometimes*^p^*	2.76	0.073	2.62–2.90	
Frequently*^q^*	2.67	0.042	2.58–2.75	
**High SES**	**29.84**			***p* < 0.001**
No	2.52	0.041	2.44–2.59	
Yes	2.69	0.067	2.56–2.82	
**Low SES**	2.21			*NS*
No	2.56	0.053	2.45–2.66	
Yes	2.65	0.071	2.52–2.79	

*NS*, non-significant.

^a^Comprised of speech and language therapists, occupational therapists, and physical therapists.

^b^Comprised of behavioral therapists, behavioral analysts, behavioral technicians, etc.

^c^Comprised of special educators, general educators, school psychologists, etc.

^d^Comprised of psychiatrists, neurologists, health practitioners, etc.

^e^Comprised of social workers, psychologists, counselors, etc.

^f^Comprised of managers, administrators, support workers, other disciplines, multiple disciplines, and unknown.

**TABLE 4:** Summary of comparisons.

	Overall^a^	Environmental modification/antecedent strategies	Behavioral analytic strategies	Cognitive strategies
Provider discipline	Medical providers < All other disciplines	Other > education, allied health, behavioral, mental health > medical	Other and behavioral > allied health, mental, educational > medical providers	Mental health > all disciplines
Provider education	NS	Master’s = Doctoral > 4 year degrees or less	Master’s = Doctoral > 4 year degrees or less	Doctoral > Master’s > 4 year degrees or less
Provider experience^b^	Most, least > middle	Most > least > middle	most > least > middle	NS
Client characteristics^c^	“Yes” High and/or low SES > Others	Sometimes ID > others “Yes” Low SES > “No” Low SES	Frequent ID > others “Yes” Low SES > “No” Low SES “Yes” High SES > “No” High SES	Awareness of ID > “Unsure” ID^d^ “Yes” High SES > “No” High SES

*NS*, non-significant predictor; contrast summary not reported.

^a^Denotes comparisons across all strategies.

^b^Years of experience working with individuals with ASD; Most = 21 + years, Middle = 11–20 years, Least = ≤ 10 years.

^c^Client-level characteristics included provider endorsement (Y/N) of working with High SES and/or Low SES clients; and working with individuals with co-occurring ID (Frequently, Sometimes, Never/Rarely, or “Unsure”).

^d^Awareness of ID includes providers who Frequently, Sometimes, or Never/Rarely worked with individuals with ID.

#### Environmental modifications/antecedent strategies

Provider familiarity with *environmental modification*s/*antecedent strategies* was predicted by: provider discipline, educational attainment, years working with individuals with ASD, and client characteristics including co-occurring ID and low SES. *Post hoc* analyses found that providers from the “other” discipline (i.e., managers, administrators, support workers, other disciplines, multiple disciplines, and unknown) category were more familiar with *environmental modifications/antecedent strategies* than all discretely-defined disciplines; medical providers were least familiar with this practice set as compared to all other disciplines. No statistically significant differences were found between non-medical providers on familiarity with *environmental modifications/antecedent strategies*.

Providers with master’s degrees reported greater familiarity with this practice set relative to providers with 4-year and less than 4-year degrees, whereas providers with doctoral degrees were only more familiar with *environmental modification*s/*antecedent strategies* than providers with less than 4-year degrees. In terms of years of experience, providers with ≥21 years of experience were the most familiar with this practice set, followed by providers with the least number of years (0–10 years) and then providers in the middle range of experience (11–20 years). Providers who sometimes work with those with co-occurring ID were generally the most likely to be familiar with this practice set, as were those who work with low SES populations.

#### Behavior analytic strategies

Provider familiarity with *behavior analytic strategies* was predicted by: provider discipline, educational attainment, years working with individuals with ASD, and client characteristics (i.e. co-occurring ID, low SES background). *Post hoc* analyses found that providers from the “other” discipline category were more familiar with *behavior analytic strategies* than the rest of the provider disciplines except for behavioral provider discipline. Providers from the behavioral discipline were more familiar with *behavior analytic strategies* than providers from the allied health, mental health, and medical disciplines. Providers from medical disciplines were the least familiar with *behavior analytic strategies*.

Providers with doctoral and master’s degrees were more familiar with this practice set than providers with 4-year degrees or less than a 4-year degree. Providers with the most experience working with individuals with ASD (≥21 years) were the most familiar with *behavior analytic strategies*, followed by the least experienced providers (0–10 years), and then middle range of experience (11–20 years or experience). Providers who worked frequently with individuals with co-occurring ID generally had more familiarity with this practice set, as did providers working with both low and high SES populations.

#### Cognitive strategies

Provider discipline, educational attainment, youth characteristics (co-occurring ID and high SES background), but not years of ASD experience significantly predicted familiarity with *cognitive strategies*. *Post hoc* analyses found that providers from mental health disciplines were the most familiar with *cognitive strategies* and were significantly more familiar with *cognitive strategies* than behavioral or medical providers.

Providers with doctoral degrees endorsed the greatest familiarity with *cognitive strategies*, followed by providers with master’s degrees, and then providers with less than a master’s degree. Finally, providers who were unsure if they provided services to youth with co-occurring ID were least likely to be familiar with *cognitive strategies* compared with providers who were sure, regardless of the frequency of such service delivery, and providers who provide services to youth with ASD from high SES backgrounds were more familiar with *cognitive strategies* compared to providers who did not.

## Discussion

This study provides the first large-scale, multidisciplinary, comprehensive evaluation of the landscape of treatment practice familiarity for youth with ASD and co-occurring anxiety, externalizing behaviors, and social skills deficits in usual care settings in the United States. Findings suggest that common treatment practices are largely familiar to practitioners, though those with medical degrees, in middle career stages, and those who did not report treating high- or low-SES clients indicated less familiarity overall. They also suggest that such practices fall into three practice sets: *environmental modification*/*antecedent strategies*, *behavior analytic strategies*, and *cognitive strategies*. This suggests that treatment practices—rather than being discrete atomistic entities—are known to the providers (and likely available to ASD communities) in contiguous groups or packages, and such groupings are intelligible to practitioners across disciplines. That is, providers who are familiar with one practice are rarely familiar with *only* that practice, and the groups of practices with which practitioners are familiar tend to “hang together” in consistent and meaningful ways.

When considering the practice sets, the *environmental modification*/*antecedent strategies* (e.g., providing choices, environmental structuring, visual supports) was the one with which providers, on average, indicated themselves to be most familiar, and also the one that explained the most variance in familiarity in general. Indeed, while the three factors explain 75% of the variance in familiarity ratings (a notably high proportion in large scale surveys such as the present one), the vast majority of that variance was explained by the 9-item *environmental modification*/*antecedent strategies* factor. This suggests that, when providers report being familiar with practices to treat youth with ASD, it is likely the case that they are either *primarily* or *also* familiar with *environmental modification*/*antecedent strategies*. In fact, familiarity with *environmental modification*/*antecedent strategies* could be considered a potential proxy for overall familiarity with treatment practices for this population. Future research may capitalize on this finding by utilizing familiarity with the nine *environmental modification*/*antecedent strategies* items to gauge general familiarity with ASD treatment in novel practitioner communities. Likewise, in training settings, introducing these strategies (particularly when there is evidence to support their effectiveness) may provide valuable inroads for training ASD treatment-naïve practitioners on potential domain-general “core elements” of treating ASD (rather than, for instance, focusing on practice elements specific only to treating specific cognitive or behavioral sequelae of ASD). Such strategy introduction represents one of numerous ways of increasing familiarity, and others are worthy of exploration based on consonance with discipline-specific learning strategies. For instance, some disciplines may respond best to case studies, while others may respond to video vignettes, provision of contact information (e.g., of local providers who utilize a given strategy), or testimonials from families and individuals describing their own experience of a given strategy.

The practice sets were largely familiar to providers who serve youth with ASD and present with varied levels of educational attainment and years of experience. The relationship between provider background and familiarity with the array of treatment practices used for this population was variable depending on the practice set. For example, medical providers reported the least familiarity with all practice sets, while mental health providers were most familiar with cognitive strategies only. The relative lack of familiarity with all presented strategies among medical providers is important to highlight given that they report a lack of confidence and training in strategies to work effectively with youth with ASD, particularly when it comes to the challenging behaviors that are often exhibited by children with ASD, including during medical visits (25). Moreover, even if medical providers themselves are not directly providing these interventions, knowledge of existing practices for the population is critical for discussing treatment recommendations with families and facilitating timely referrals to appropriate services. To be sure, pediatricians and primary care providers represent the “front line” of providers who are among the first to raise concerns about ASD and facilitate referrals to additional services (26). As such, efforts to increase medical providers’ awareness and familiarity with behavioral approaches may result in improved care within the medical visits, as well as more efficient and effective care coordination and treatment access.

It is notable that having a doctoral degree did not appear to confer consistent advantages in terms of familiarity with *environmental modification*/*antecedent strategies* and *behavior analytic strategies* beyond a 4-year degree or less. Indeed, master’s level and doctoral level clinicians reported similar familiarity with *environmental modification*/*antecedent strategies* and *behavior analytic strategies*, whereas *cognitive strategies* were more familiar to doctoral-level providers than masters-level providers and all other groups, suggesting greater exposure to these strategies in doctoral-level training and/or continuing education. The adaptation of cognitive behavioral therapy (CBT) to individuals with ASD represents a relatively new treatment approach (27, 28); increased awareness of how cognitive strategies can be delivered to individuals with ASD might be more salient among those highly connected to research settings (e.g., doctoral level clinicians) (29, 30). In addition, while mid-career practitioners indicated the least familiarity overall, the most experienced providers (>21 years’ experience), followed by the least experienced providers (<11 years’ experience) endorsed greatest familiarity with *environmental modification*/*antecedent* and *behavior analytic strategies*, but not *cognitive strategies*, highlighting both the longer history of these strategies in the field, and their more frequent inclusion in introductory training settings.

Client-level characteristics, such as co-occurring ID and client SES, were also associated with providers’ endorsement of familiarity, although this too varied by specific practice set. Indeed, those providers who frequently or sometimes treated youth with co-occurring ID endorsed greater familiarity with the *environmental modification*/*antecedent strategies* and *behavior analytic strategies* practice sets relative to providers who never or rarely treated these youths. Notably, for the *cognitive strategies* practice set, the only familiarity differences were that providers who were unsure of whether they treated youths with ID reported less familiarity than the other providers, perhaps because these providers work in roles (e.g., supervisory) that keep them at a greater distance from individual participants and strategies used for them. That is, if a provider does not know whether they are serving ay children with ID, it may be because they are not directly interacting with those children (and may be in a supervisory role). Similarly, some practices may not require the practitioner to ascertain formal cognitive ability in the children they serve; for instance, if someone uses the “choice making” practice, they may do so regardless of whether the child they are serving can speak or not. Thus, future work should better ascertain this unusual response type and what it may denote.

For SES, those who reported treating high *and/or* low SES clients reported the greatest overall familiarity, though this pattern was more nuanced at the level of the practice sets. Specifically, providers who endorsed treating high SES clients reported greater familiarity with *behavior analytic* and *cognitive strategies*, perhaps because they can be provided in more individualized formats; conversely, providers who endorsed treating low SES clients reported greater familiarity with *environmental modification*/*antecedent* and *behavior analytic* strategies, perhaps because they are more amenable to setting-level implementation when resources are low.

### Limitations

Several limitations warrant comment. First, although data were collected across five geographically distinct sites, the participant sample may not be representative of providers delivering services to youth with ASD across the entire United States. Next, this study examined only practices relevant to all three indicated referral problems, and therefore does not represent familiarity with practices specific *only* to a given one (e.g., video modeling for social skills). Another limitation is that our data reflect providers’ self-reported familiarity with intervention strategies as described in the UCAS—they may recognize them if exposed to them directly (e.g., via video). Further, it cannot be known whether providers correctly identified themselves as being familiar—that is, they may have stated awareness of a given practice, but perhaps might have misunderstood what was intended. Relatedly, the cell size for the group who indicated they are “unsure/don’t know” if they treat intellectual disability was small, and while this study did employ an analytic approach that mitigates site-specific effects, many in this cell happened to be from a single site. In addition, our study did not delineate between which practices or practice sets are evidence-based practices, as determined by various criteria (31, 32). Given the vast scope of practices used by practitioners from a range of professional backgrounds, the current study sought to broadly capture the landscape of familiarity of practices *in general*, regardless of whether the practice may be evidence-based. It will be imperative that future studies delineate between evidence-based practices and those practices with less empirical support, as the delivery of evidence-based practices to youths—especially those with ASD—represents a critical need-to-practice gap (33).

## Conclusion

Overall, this study found that common treatment practices used in intervention with youth with ASD fall into three coherent familiarity practice sets, with several provider- client- and setting-related characteristics predicting differences. It also suggests that *environmental modification*/*antecedent strategies* may represent the most common or “core” practice elements representing familiarity with ASD treatment practices. Given the importance of familiarity for both direct service provision and service referral, variability of provider familiarity reveals several needs. First, differences in familiarity highlight the need for knowledge of practices to be broadly and regularly disseminated to providers, especially for those providers who facilitate referral to services (e.g., medical providers). Second, provider familiarity of strategies could also be improved through coordinated multidisciplinary treatment approaches and integrated care settings. Finally, training programs and their curricula—regardless of discipline—play an essential role in increasing provider familiarity with and consequent access to interventions that are used with ASD youth. These findings provide guidance on each of these fronts, offering the first mapping of familiarity with treatment practices for youth with ASD in the United States across disciplines.

## Data availability statement

The raw data supporting the conclusions of this article will be made available by the authors, without undue reservation.

## Ethics statement

The studies involving human participants were reviewed and approved by the Office of Research Compliance at Stony Brook University, the Institutional Review Board (IRB) at St. John’s University, the IRB at Drexel University, the IRB at Rush University’s Office of Research Affairs, and the IRB at San Diego State University. The patients/participants provided their written informed consent to participate in this study.

## Author contributions

ML contributed to study conceptualization, methodology, project multisite administration, recruitment, leading statistical analyses and interpretations of results, and writing the original text. CB, AS, JT, PF, and EL contributed to statistical analysis and writing – original draft. CK, LM, AW, and EC contributed to project administration, recruitment, and data collection and review/editing of the manuscript. AD contributed to project administration and data collection, statistical analyses and results interpretation, writing the original text, and edited manuscript content and format. All authors contributed to the article and approved the submitted version.
